# Association between interleukin-12 +1188A/C polymorphism and the risk of tuberculosis: A meta-analysis

**DOI:** 10.4314/ahs.v23i2.15

**Published:** 2023-06

**Authors:** Pengcheng Wang, Jiadan Liao

**Affiliations:** 1 Department of Tuberculosis,Affiliated Hangzhou Chest Hospital, Zhejiang University School of Medicine, Hangzhou, China; 2 Department of Cardiology, the Third Affiliated Hospital of Zhejiang Chinese Medical University, Hangzhou, China

**Keywords:** IL-12, tuberculosis, meta-analysis

## Abstract

**Background:**

The role of *interleukin-12(IL-12)* gene +1188A/C polymorphism has been indicated in the progression of tuberculosis (TB). Nevertheless, the outcomes remain controversial.

**Objectives:**

This meta-analysis focused on examining the association between *IL-12* +1188A/C polymorphism and TB.

**Methods:**

EMBASE and PubMed databases were searched to identify relevant studies for retrospective analysis. Then, a random- or fixed-effects model was utilized to calculate the combined odds ratios (ORs) as well as corresponding 95% confidence intervals (CIs).

**Results:**

A total of 9 related articles were discovered. The result of meta-analysis showed that *IL-12* +1188A/C polymorphism did not show significant correlation with TB risk among the total population (C vs A: OR =0.66, 95% CI=0.41-1.05; AC vs AA: OR = 1.28, 95% CI =0.90-1.82; CC vs AA: OR =0.95, 95% CI=0.80-1.14; recessive model: OR =1.00, 95% CI = 0.75-1.34; dominant model: OR =1.07, 95% CI =0.94-1.21). As discovered from subgroup analyses based on race and HWE, this polymorphism did not show relationship with TB risk.

**Conclusion:**

The present findings indicated that *IL-12* +1188A/C polymorphism was not related to the risk of TB.

## Introduction

Tuberculosis (TB) has become a major public health issue worldwide, in particular among the developing countries. It is reported by statistics that, the annual mortality and morbidity rates of TB are as high as 1.5 and 9 million, separately^1^. However, its exact pathogenic mechanism and etiology remain unclear. As predicted by the World Health Organization (WHO), TB infection affects around 1/3 of the world's population, while just 10% among the infected people finally have clinical disorders^2^. Generally speaking, the risk factors for Mycobacterium tuberculosis (MTB) infections include living in the developing countries, obesity, diabetes, malnutrition, and smoking. But TB represents a complicated disorder with unclear etiology. Moreover, accumulative studies suggest that different genetic factors are related to TB susceptibility in the host^3^. IL-12, the heterodimeric protein with the molecular weight of 70-kDa, contains 2 subunits (p35, p40) that are connected via the disulfide bond^4^. IL12-p40 plays an essential role in protecting against MTB mediated by IFN, and it is also a key factor involved in Th1 cell differentiation^5^. Cases having genetic defects related to the IL-12 signal transduction are associated with a higher risk of bacillus Calmette-Guerin and environmental mycobacteria infection^6^. A mutation is detected in the 3′ untranslated region (UTR) +1188A/C(rs3212227), which has been suggested to regulate gene level. Such polymorphism in human beings is discovered to induce a severe hereditary impaired immunity to MTB infection, and risks of autoimmune disorder and type1 diabetes mellitus (T1DM)^7^.

Many articles report the relationship of TB risk with *IL-12* +1188A/C polymorphism, but no consensus has been reached so far. One case-control study is probably insufficient for the comprehensive illustration of the complex relationship due to the small sample size. Meta-analysis has been the helpful method to analyse complicated data from case-control studies were somewhat limited by small sample sizes and represent lower statistical power^8^. This meta-analysis focused on investigating the relationship of *IL-12* +1188A/C polymorphism with TB risk through conducting a meta-analysis of all relevant articles published to date.

## Materials and methods

### Search Strategy

We searched PubMed and EMBASE databases to identify related studies (from inception to May 1^st^, 2021), by the following search terms “IL-12” or “+1188A/C” or “rs3212227” and “tuberculosis”. Only studies published in English language were enrolled. We evaluated the relevant literatures to search all the qualified articles. Their reference checklists were also manually searched for identifying possible literature. For repeated studies, just the study that had most data was enrolled. The protocol is registered in PROSPERO (ID:350023).

### Inclusion and Exclusion

Studies consistent with the following inclusion criteria were enrolled into this work:1) related case-control literature including TB patients as well as the normal subjects; 2) studies on the association between *IL-12* +1188A/C and TB risk; 3) studies including enough genotype data to be extracted. Studies meeting the following exclusion criteria would be excluded: 1) non-case-controlled studies; 2) case reports, meta-analyses, reviews; 3) articles that did not have enough information to be collected.

### Data extraction

Relevant data were obtained, including first author, race, country, publication date, case and control numbers, *IL-12* +1188A/C polymorphism frequency, as well as Hardy-Weinberg Equilibrium (HWE) test of control group.

### Evaluation of study quality

The authors assessed the methodological quality of each included article using the Newcastle-Ottawa quality assessment scale (NOS). An ultimate score of 6 stars or more was regarded as a high-quality study.

### Statistical analysis

Firstly, this study conducted HWE test of control group in each single study by chi-square test. Then, we assessed the strength of association of the *IL-12* +1188A/C polymorphism with TB susceptibility through calculating OR and 95% CIs. Four different ORs were calculated: heterozygote comparison (AC vs. AA), homozygote comparison (CC vs. AA), recessive model (CC vs. AC+AA) and dominant model(AC+CC vs. AA). In the meantime, possible differences across the collected articles were analysed through I^2^ test, where I^2^>50% suggested differences among the collected research, and a random effects model was selected accordingly, and the fixed effects model was employed otherwise. Moreover, HWE-stratified subgroup analyses were conducted as well. For assessing the stability of our results, we performed sensitivity test through eliminating each article each time to examine its influence on the pooled ORs. Besides, we drew the funnel plot to assess publication bias. The R 3.33 software meta-package was used in this meta-analysis.

## Results

### Characteristics of Included Studies

**Figure 1:**
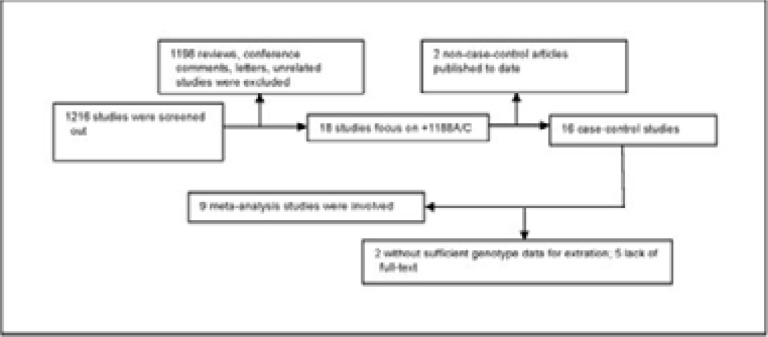
The flow diagram of included/excluded studies

In line with our search strategy, we searched diverse databases and obtained altogether 1216 studies. Figure 1 shows more details about the study search flowchart. In the next stage all of the abstracts were scanned, which eliminated 1198 studies (according to review of titles, keywords and abstracts), leaving 18 for subsequent analyses. Later, we further eliminated 9 studies due to the lack of full-text (n=5), lack of genotype information (n=2) and non-case-control articles (n=2). Eventually, 9 eligible articles satisfying our pre-set inclusion criteria were enrolled^8^-^16^. Table 1 displays the study features. According to the NOS for case-control studies, the overall scores of the included studies ranged from six to eight stars. All studies were defined as high-quality.

**Table 1:** Characteristics of the included studies for meta-analysis

Study included	Year	Area	Race	Cases/Controls	Allele for cases		Allele for controls		Genotypes for cases			Genotypes for controls			HWE test

					A	C	A	C	AA	AC	CC	AA	AC	CC	
Ma (White)	2003	USA	Caucasian	106/117	167	45	199	35	65	37	4	85	29	3	0.78
Ma (African)	2003	USA	African	186/167	233	139	224	110	77	79	30	75	74	18	0.97
Tsao	2004	China	Asian	516/514	612	420	559	469ee	166	280	70	143	273	98	0.11
Selvaraj	2008	India	Asian	160/183	184	136	219	147	57	70	33	70	79	34	0.17
Naslednikova	2009	Russia	Caucasian	78/82	107	49	113	51	34	39	5	41	31	10	0.29
Wang	2010	China	Asian	518/526	590	446	620	432	168	254	96	178	264	84	0.40
Arji	2011	Morocco	African	337/204	452	222	286	122	152	148	37	104	78	22	0.21
Mahmut	2013	Turkey	Caucasian	84/110	124	44	160	60	47	30	7	58	44	8	0.93
Shruthi	2016	India	Asian	360/200	391	329	203	197	108	175	77	66	71	63	0.00
Olga	2021	Mexico	Caucasian	14/138	4	24	92	184	0	5	9	0	92	46	0.00

HWE, Hardy–Weinberg equilibrium.

### Meta-analysis results

**Figure 2:**
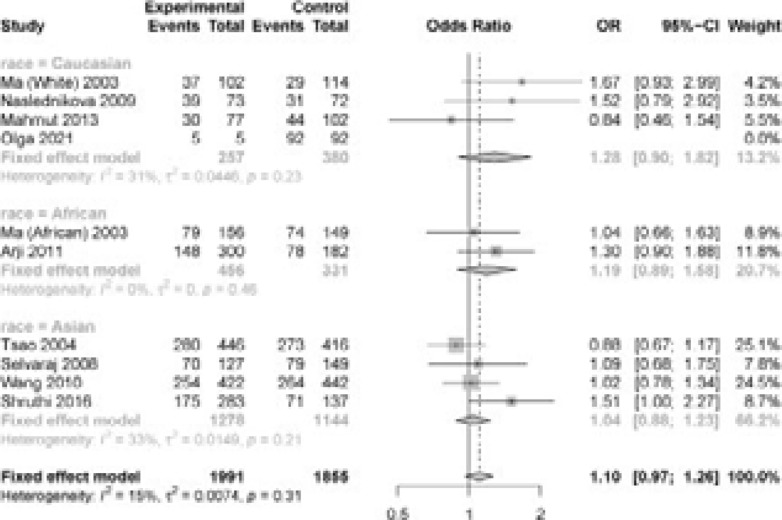
Forest plot for meta-analysis of the association between the IL-12 +1188A/C polymorphism and TB risk under dominant model

According to Table 2, *IL-12* +1188A/C polymorphism was not significantly related to TB in each genetic model (Figure 2, C vs A: OR =0.66, 95% CI=0.41-1.05; AC vs AA: OR = 1.28, 95% CI =0.90-1.82; CC vs AA: OR =0.95, 95% CI=0.80-1.14; recessive model: OR =1.00, 95% CI = 0.75-1.34; dominant model: OR =1.07, 95% CI =0.94-1.21). Based on race-stratified subgroup analysis, there was no obvious relationship in Caucasians, Asians and Africans. According to HWE-stratified subgroup analysis, *IL-12* +1188A/C polymorphism was not significantly related to TB susceptibility.

**Table 2:** Summary of different comparative results

Variables	N	C vs A		CC vs AA		AC vs AA		Dominant model		Recessive model	

		OR (95%CI)	model	OR (95%CI)	model	OR (95%CI)	model	OR (95%CI)	model	OR (95%CI)	model
**Total Ethnicity**	10	0.66 (0.41-1.05)	R	0.95(0.80-1.14)	F	1.28(0.90-1.82)	F	1.10(0.97-1.26)	F	1.00(0.75-1.34)	R
Asians	4	0.85 (0.69-1.04)	F	3.57(0.37-34.54)	R	1.04(0.88-1.23)	F	1.04(0.88-1.23)	F	0.85(0.59-1.22)	R
Caucasians	4	0.34 (0.07-1.56)	R	0.96(0.48-1.92)	R	1.19(0.89-1.58)	F	1.28(0.90-1.82)	F	1.30(0.56-3.02)	R
Africans	2	1.05 (0.90-1.23)	R	1.34(0.86-2.07)	R	1.04(0.88-1.23)	F	1.19(0.89-1.58)	F	1.25(0.82-1.89)	F
**HWE**												
Yes	8	0.65 (0.37-1.12)	R	1.00(0.82-1.22)	F	1.06(0.92-1.22)	F	1.06(0.93-1.21)	F	0.98(0.82-1.18)	F
No	2	0.70 (0.21-2.31)	R	0.75(0.48-1.17)	F	1.51(1.00-2.27)	F	1.15(0.79-1.67)	F	1.34(0.23-7.82)	R

N: number; CI: confidence interval; OR: odds ratio; R: random effects model; F: fixed effects model.

### Sensitivity Analysis

**Figure 3:**
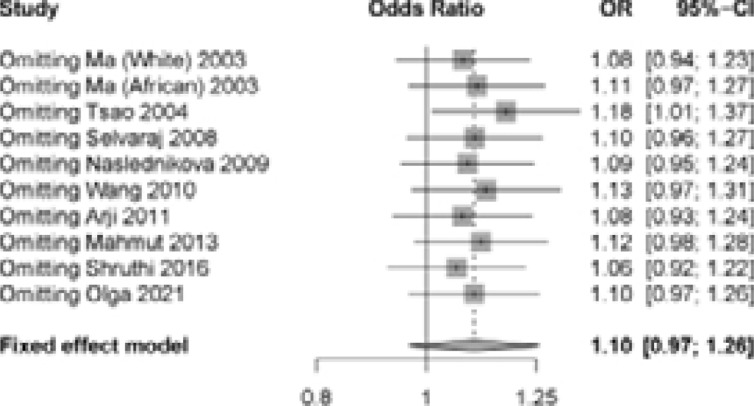
Sensitivity analysis between the IL-12 +1188A/C polymorphism and TB under dominant model

For the pooled results, their statistical significance remained unchanged after eliminating one article, which indicated that our result was statistically significant (Figure 3).

### Publication bias

**Figure 4:**
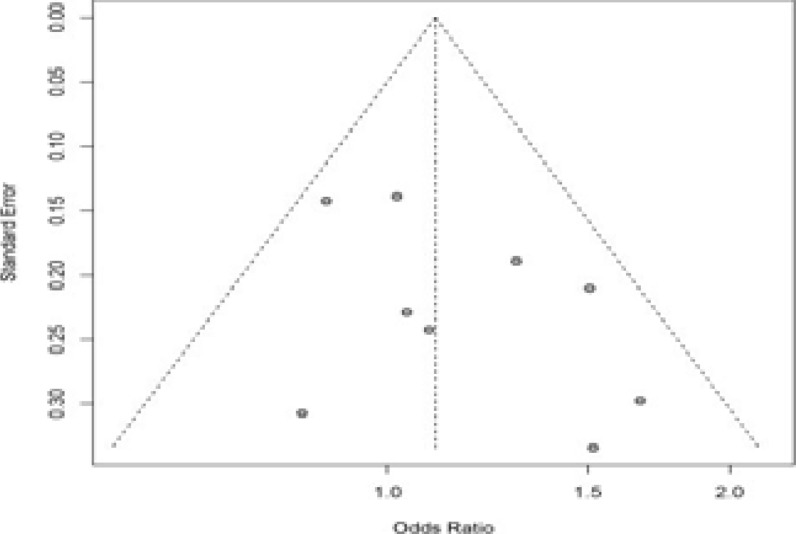
Begg's funnel plot analysis to detect potential publication bias for the IL-12 +1188A/C polymorphism under dominant model

We drew the Egger's funnel plot for assessing the possible publication bias among the existing studies. No obvious asymmetry was observed in the funnel plot (Figure 4).

## Discussion

TB, a kind of MTB-induced infectious disorder, accounts for a major reason for death resulting from one individual infectious agent globally^17^. Typically, the innate immunity in the host plays a vital role in activating adaptive immunity and initial anti-MTB defence. Several studies have reported the possible relationship of *IL-12* +1188A/C polymorphism with TB risk. However, the pathogenesis of TB is not clearly understood and remains to be elucidated, which might be mostly due to the small sample size in individual case-control research. As a result, the present meta-analysis focused on summarizing the relationship between *IL-12* +1188A/C polymorphism and risk of TB.

To our knowledge, for the first time, we investigated this issue in a meta-analysing way via the quantitative evaluation of the relationship of *IL-12* +1188A/C polymorphism with TB risk. Notably, we efficiently collected eligible researches via both electronical and manual searching approach, rendering the inclusion of almost all eligible studies. In our study, night case-control researches were enrolled, involving 2359 patients as well as 2241 healthy controls. Overall, *IL-12* +1188A/C polymorphism is not the significant risk factor in TB risk. With regard to the difference of the environment in which they lived in, we perform a ethnicity-specific subgroup analysis, and the result failed to reveal any correlation of this polymorphism with TB susceptibility. Allele distribution deviations from HWE also resulted in the heterogeneity between studies. As revealed by our subgroup analysis by restricting our meta-analysis to HWE-consistent studies, our data were robust. There was no evident evidence of publication bias. Similar to additional disorders, TB is induced by the complex associations between some genes and between genes and the environment. Such associations should be further investigated in the future.

Many previous studies have shown that genes are related to anti-TB treatment. Xin et al found that xanthine oxidase gene polymorphisms might contribute to anti-TB drug-induced liver injury^18^.Achmad et al found that NAT2 Gene rs1041983 is associated with anti-Tuberculosis drug induced hepatotoxicity^19^. In addition, previous study showed that CYP2B6 G516T polymorphism is a key predictor of the therapeutic response to treatment in TB patients^20^.

Certain limitations must be noted in this meta-analysis. Firstly, many of our enrolled articles just provided the data of *IL-12* +1188A/C polymorphism with TB susceptibility, but the accurate adjusted ORs including other data like family history, age or environmental factors, were not determined. Secondly, our enrolled articles on *IL-12* +1188A/C polymorphism only included few samples, which might result in the poor statistical power, in particular for subgroup analyses. Finally, this study did not investigate the impacts of interactions between genes and between genes and the environment.

To sum up, we found that the *IL-12* +1188A/C polymorphism was not associated with TB susceptibility. Additional large-sample, high-quality, and well-designed studies focused on this SNP should be carried out to confirm their involvement in the development of TB.
